# Supplementary material for: piRNA loading triggers MIWI translocation from the intermitochondrial cement to chromatoid body during mouse spermatogenesis
Source: Nat Commun. 2024 Mar 15;15:2343. doi: 10.1038/s41467-024-46664-3 (PMC10943014; doi:10.1038/s41467-024-46664-3)
Supplement: Supplementary file 1 — Supplementary information [file 41467_2024_46664_MOESM1_ESM.pdf]

Supplementary Information

piRNA loading triggers MIWI translocation from the intermitochondrial cement to chromatoid body during mouse spermatogenesis

Huan Wei, Jie Gao, Di-Hang Lin, Ruirong Geng, Jiaoyang Liao, Tian-Yu Huang, Guanyi Shang, Jiongjie Jing, Zong-Wei Fan, Duo Pan, Zi-Qi Yin, Tianming Li, Xinyu Liu, Shuang Zhao, Chen Chen, Jinsong Li, Xin Wang *, Deqiang Ding * and Mo-Fang Liu*

***Correspondences**

mfliu@sibcb.ac.cn (M.-F.L.); dingdeqiang@tongji.edu.cn (D.D.); wx@ucas.ac.cn (X.W.)

The PDF file includes

Fig. S1 | The construction of piRNA loading-deficient *Miwi*$^{YY/YY}$ and *Miwi*$^{YK/YK}$ mice.

Fig. S2 | piRNA loading-deficient mutations in MIWI barely impair the production of MILI-interacting piRNAs in *Miwi*$^{YY/YY}$ and *Miwi*$^{YK/YK}$ mice.

Fig. S3 | Immunostaining of MILI and MVH in testis sections from *Miwi* mutant mice.

Fig. S4 | Validation of the specificity anti-MIWI^{unloaded} antibody.

Fig. S5 | TDRD6 depletion does not affect MIWI and piRNA expression in mice.

Fig. S6 | TDRD6 depletion impairs MIWI integration into the CB during male germ cell differentiation in mice.

Fig. S7 | Loss of piRNA-loading ability impairs MIWI stability in mouse testes.

Table S1 Oligonucleotides and antibodies used in this study.

a

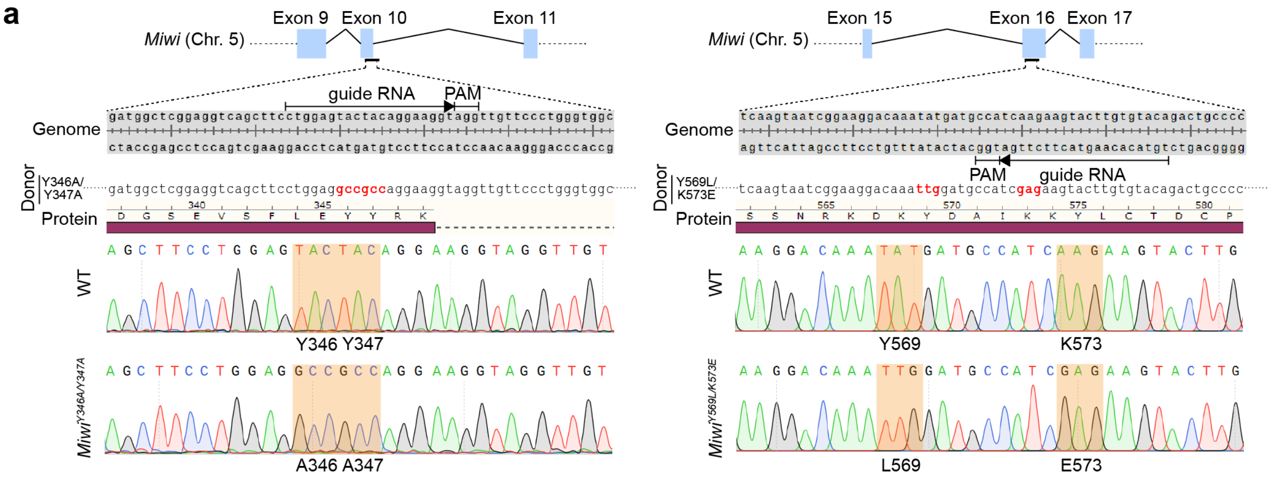

b

Off-targets of sgRNA-YY			
**Location**	**Number of mismatches**	**Sequence (including mismatches)**	**Genomic location**
chr1:36871688	3	CTGtAGTAgTACAGGAAGGaGGG	Intergenic
chr1:64043468	3	CCAACCTTCCTGggGTACTaCAG	Klf7 intron
chr10:62817828	3	CCTAaCcTCCTGcAGTACTCCAG	Tet1 intron
chr12:102221030	3	CCAAaCTTCCTGcAGcACTCCAG	Slc24a4 intron
chr13:54157474	3	CTGGAGTACTgCAGGgAGtTTGG	Intergenic
chr18:56786808	3	CCCAtCTTCCTGgAGTACgCCAG	Marchf3 intron
chr19:51242013	3	CCTACCTTCCTGTAGaACTCagG	Intergenic
chr3:93029764	3	CTGtAGaACTACAGGgAGGTGGG	Intergenic
chr3:126522082	3	CaGcAtTACTACAGGAAGGTAGG	Intergenic
chr4:55620229	3	CCTcCCTTCCTGTAGTtCTCCAt	Gm12506 intron
chr5:128744368	0	CTGGAGTACTACAGGAAGGTAGG	On-target Miwi exon
chr7:30444313	3	CCCACCTgtCTGcAGTACTCCAG	Aplp1 exon
chr7:63482384	2	CCTACCaTCCTGTAGTAgTCCAG	Otud7a intron
chr7:73757776	3	CTGGAGTACaAgAGGAAGcTGGG	Fam174b intron
chr8:22886287	3	CTGGAcTAtTACAaGAAGGTGGG	Kat6a intron
chr8:91180571	2	aTGaAGTACTACAGGAAGGTTGG	Intergenic
chr8:127757626	3	CTGGAGTACTtgAGGAAaGTGGG	Intergenic
chr9:23035036	3	CTGtAGTAaaACAGGAAGGTCGG	Gm36743 intron
chrX:21169744	3	CTGGAGgACTACAGGtAGGaAGG	Gm5932 intron

Off-targets of sgRNA-YK			
**Location**	**Number of mismatches**	**Sequence (including mismatches)**	**Genomic location**
chr16:23090100	3	CCCTCAAGAAGcACTTGcaTACA	Intergenic
chr18:37418447	3	CCCTCAAGAcaTACTTaTGTACA	Gm37013 intron
chr18:48649378	3	CCTTCAAGAAGTgagTGTGTACA	Intergenic
chr18:51909688	3	CCTTCAAtAAGTACaTGTGcACA	Intergenic
chr4:31012321	3	CCCTCAAGAAGTACTTtTGTAag	Intergenic
chr5:128749889	0	CCATCAAGAAGTACTTGTGTACA	On-target Miwi exon
chr7:46562558	3	TGTcCACAAGTAacTCTTGAGGG	Sergef intron
chrX:50467726	3	TGTACAgAAGTAaTTgTTGATGG	Intergenic

Fig. S1 | The construction of piRNA loading-deficient *Miwi*$^{YY/YY}$ and *Miwi*$^{YK/YK}$ mice. a schematic diagram illustrating the construction of *Miwi*$^{YY/YY}$ (left) and *Miwi*$^{YK/YK}$ mutant mice (right) using CRISPR/Cas9. Top, genomic structure of *Miwi* and sequences of guide RNA and donors; bottom, Sanger sequencing of genotyping PCR products amplified from the guide RNA-targeted area to confirm the mutant alleles. **b** Detection of potential off-target effects of the guide RNAs in *Miwi*$^{YY/YY}$ and *Miwi*$^{YK/YK}$ mutant mouse genome by PCR amplification and Sanger sequencing.

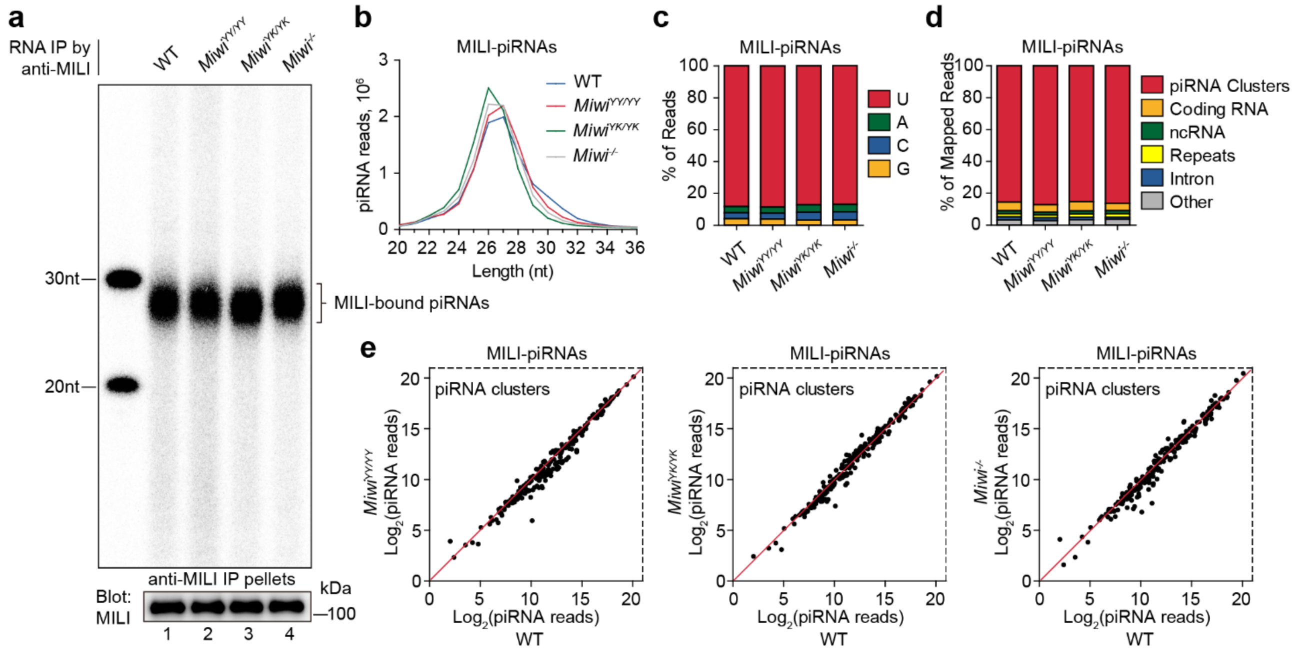

Fig. S2 | piRNA loading-deficient mutations in MIWI barely impair the production of MILI-interacting piRNAs in *Miwi*$^{YY/YY}$ and *Miwi*$^{YK/YK}$ mice. a RNA co-IP assay of MILI interacting-piRNAs (top) in wildtype (lane 1), *Miwi*$^{YY/YY}$ (lane 2), *Miwi*$^{YK/YK}$ (lane 3) and *Miwi*$^{-/-}$ testes (lane 4), with anti-MILI IB as a loading reference (bottom). **b-e** Comparison of length distribution (**b**), 1U bias feature (**c**), genomic annotation (**d**), and piRNA cluster distribution (**e**) of MILI associated-piRNAs in wildtype, *Miwi*$^{YY/YY}$, *Miwi*$^{YK/YK}$ and *Miwi*$^{-/-}$ testes. Result shown in **a** is representative of three independent experiments, and small RNA-seq experiments shown in **b-e** with two replicates. Source data are provided as a Source Data file.

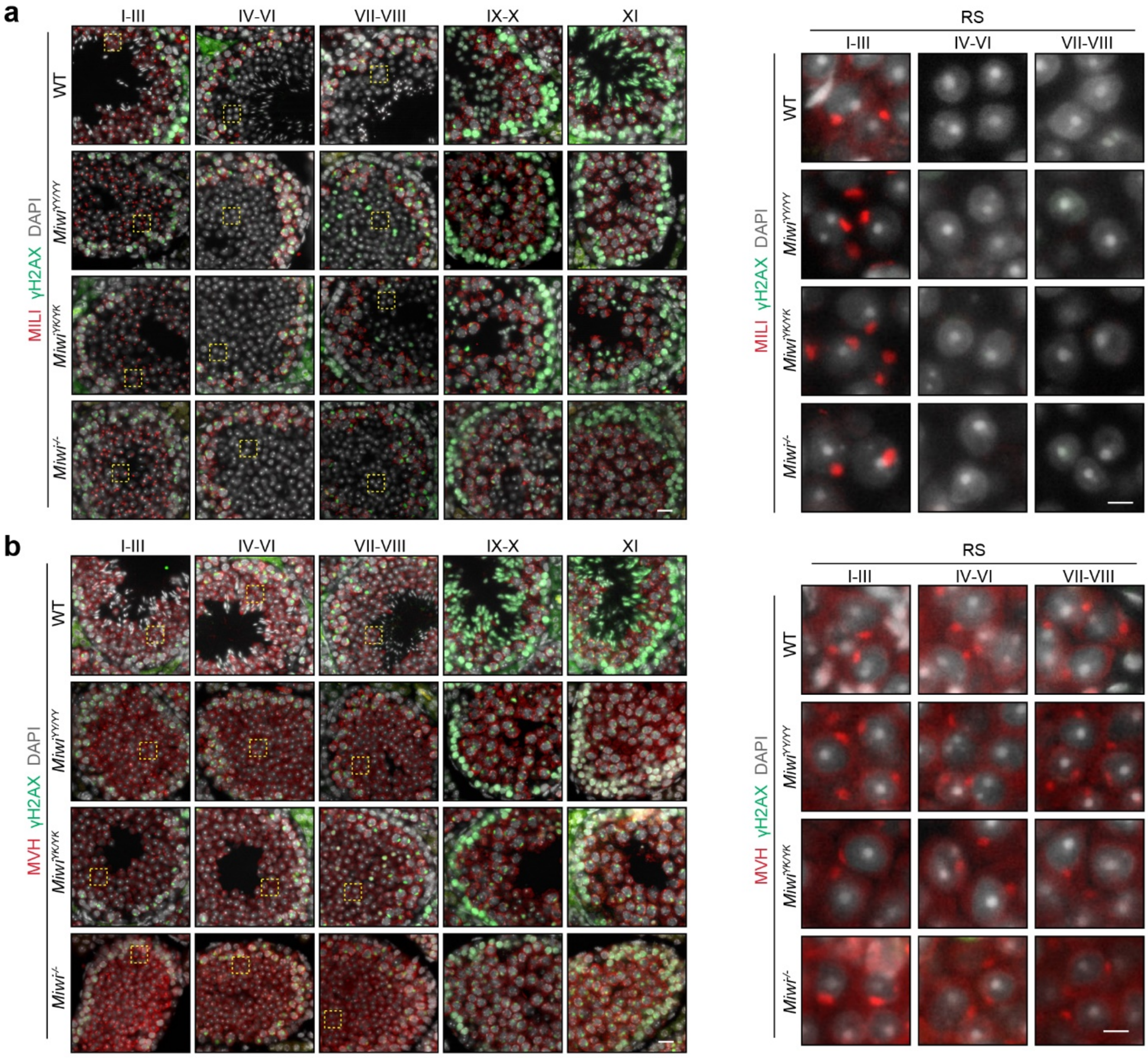

Fig. S3 | Immunostaining of MILI and MVH in testis sections from *Miwi* mutant mice. a, b Immunostaining of MILI (**a**, red) and MVH (**b**, red) on testis sections from adult wildtype, $Miwi^{YY/YY}$, $Miwi^{YK/YK}$ and $Miwi^{-/-}$ mice using regular microscopy. Left: representative staining images of indicated mouse testis sections, scale bar, 20 μm; right, the enlargement of yellow framed regions, scale bar, 5 μm. The developmental stages of spermatocytes and spermatids were distinguished according to γH2AX (green) and DAPI (greyscale) staining. RS, round spermatids. Results shown are representative of three independent experiments.

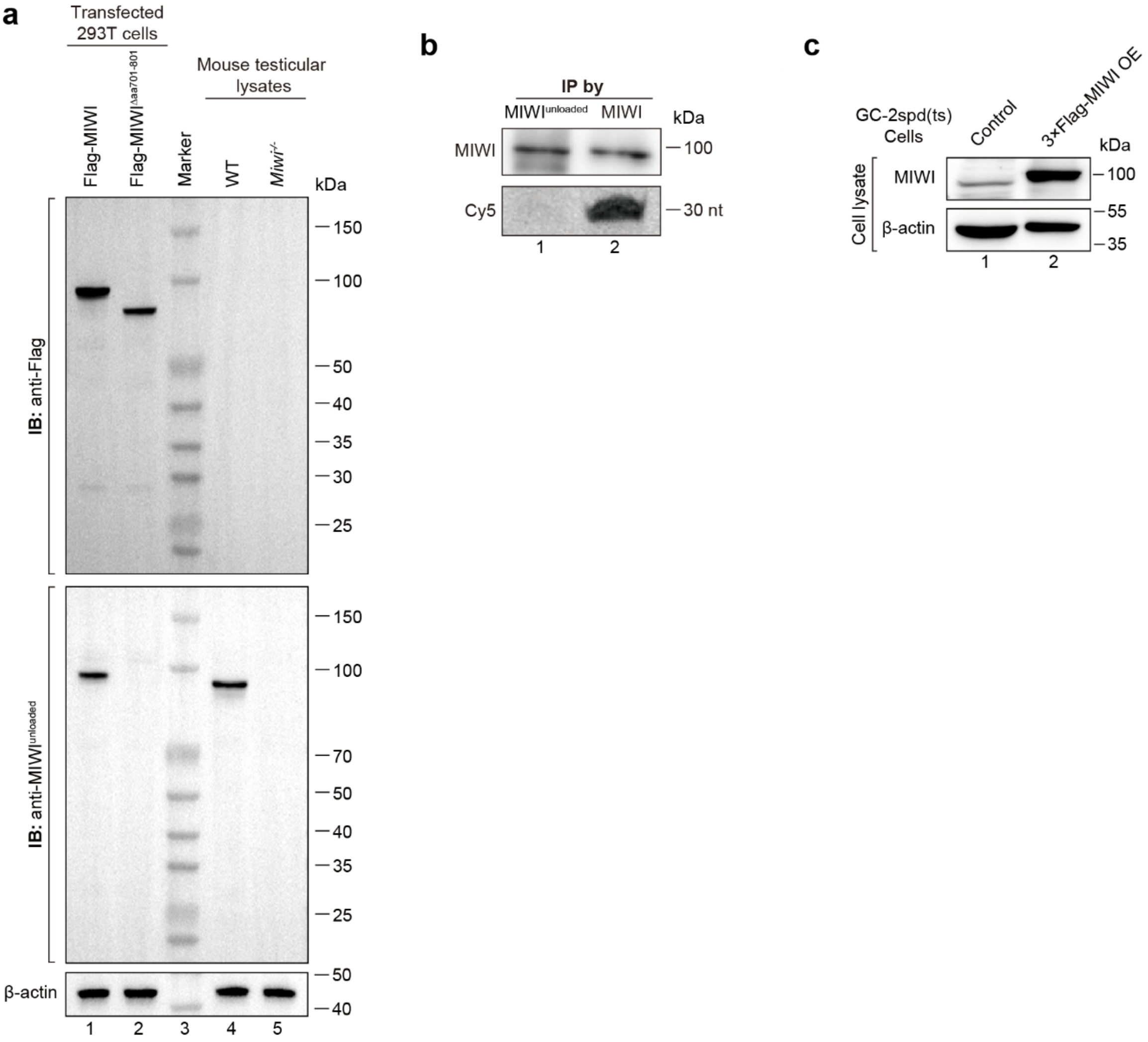

Fig. S4 | Validation of the specificity anti-MIWI^{unloaded} antibody. a Western blotting of MIWI protein using anti-MIWI^{unloaded} antibody in Flag-tagged wildtype MIWI or epitope-deleted MIWI$^{\Delta aa701-801}$ mutant-transfected HEK293T cells (lanes 1 and 2), and adult wildtype or *Miwi*$^{-/-}$ testes (lanes 4 and 5), with β-actin serving as a loading control. **b** Anti-MIWI^{unloaded} preferably pulled down piRNA-unloaded MIWI in Flag-MIWI-transfected HEK293T cells. RNA co-IP assays using anti-MIWI^{unloaded} and control anti-MIWI antibodies in cell lysate incubated with Cy5-labeled piRNA, with anti-MIWI IB as loading references. **c** Western blotting of MIWI protein in parental and Flag-tagged MIWI-stable-expressed GC-2spd (ts) cells, with β-actin serving as a loading control. Results shown are representative of three independent experiments. Source data are provided as a Source Data file.

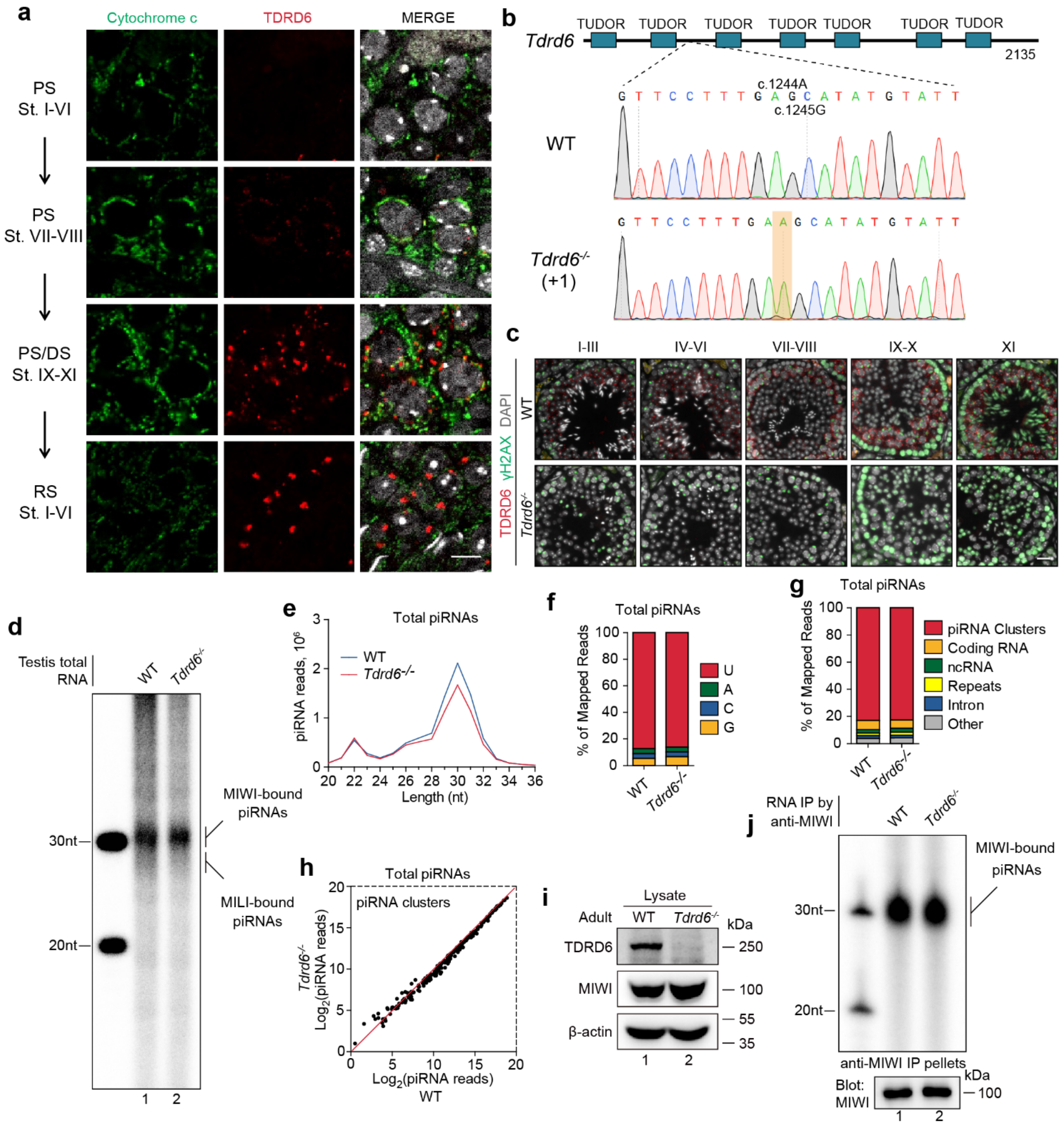

Fig. S5 | TDRD6 depletion does not affect MIWI and piRNA expression in mice. a Co-immunostaining of Cytochrome c (green) and TDRD6 (red) on testis sections from adult wildtype mice using confocal microscopy, with nuclei counterstained by DAPI (greyscale). Scale bar, 10 µm. **b** A schematic diagram illustrating the construction of *Tdrd6*$^{-/-}$ mutant mice using CRISPR/Cas9. Top, genomic structure of *Tdrd6*; bottom, Sanger sequencing of genotyping PCR products amplified from the guide RNA-targeted area to confirm the mutant allele. **c** Immunostaining of TDRD6 (red) on testis

sections from adult wildtype and *Tdrd6*$^{-/-}$ mice using regular microscopy. The developmental stages were distinguished according to γH2AX (green) and DAPI (greyscale) staining. Scale bar, 20 μm. **d** Detection of piRNA expression in adult wildtype (lane 1) and *Tdrd6*$^{-/-}$ testes (lane 2). **e-h** Comparison of length distribution (**e**), 1U bias feature (**f**), genomic annotation (**g**), and piRNA cluster distribution (**h**) of piRNAs in wildtype and *Tdrd6*$^{-/-}$ testes. **i** Western blotting of TDRD6 and MIWI proteins in adult wildtype and *Tdrd6*$^{-/-}$ testes, with β-actin serving as a loading control. **j** RNA co-IP assay of MIWI-interacting RNAs (top) from adult wildtype and *Tdrd6*$^{-/-}$ testes, with anti-MIWI IB as loading references (bottom). Results shown in **b-d**, **i** and **j** are representative of three independent experiments, and small RNA-seq experiments shown in **e-h** with two replicates. Source data are provided as a Source Data file.

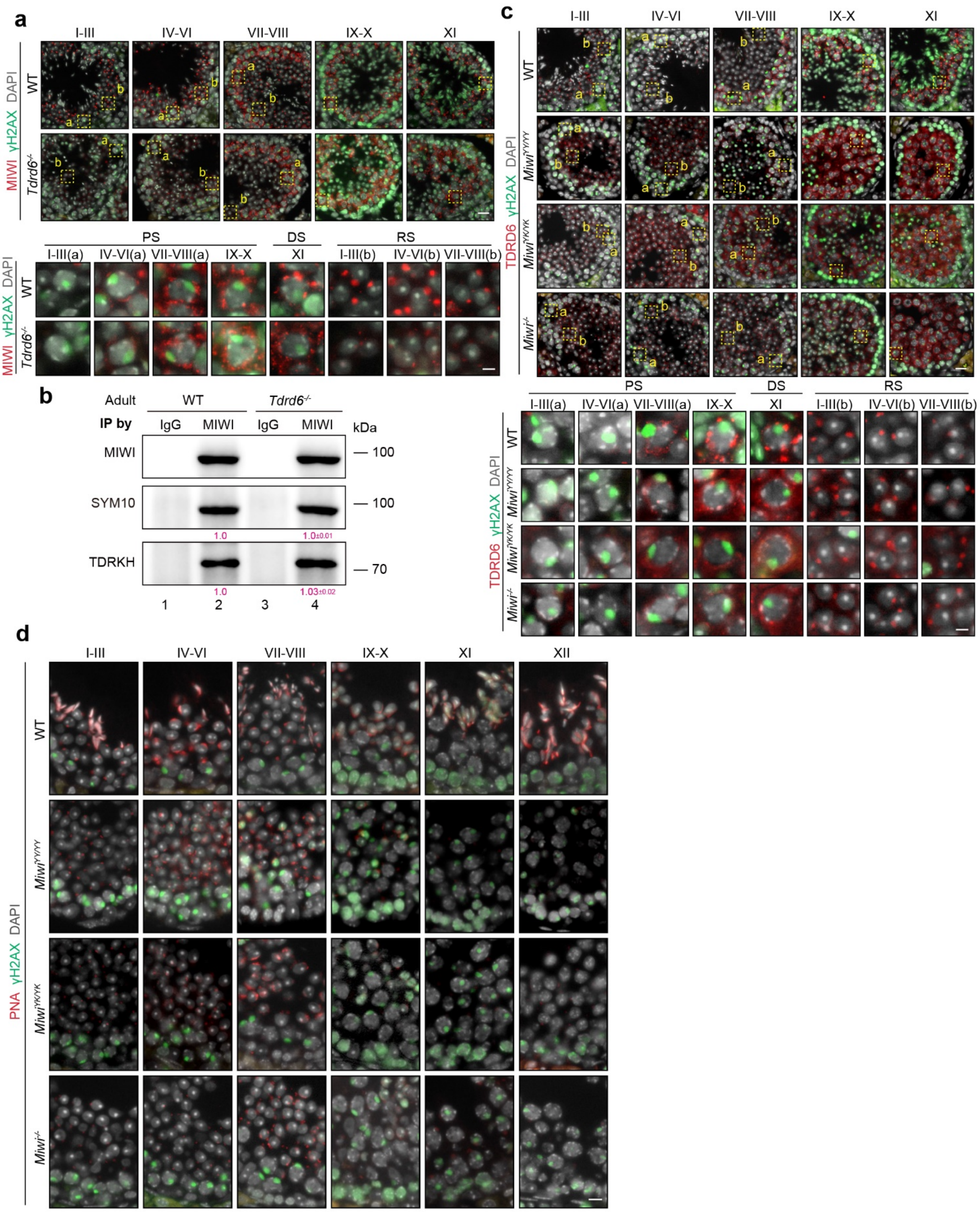

Fig. S6 | TDRD6 depletion impairs MIWI integration into the CB during male germ cell differentiation in mice. a Immunostaining of MIWI (red) on testis sections from adult wildtype and *Tdrd6*$^{-/-}$ mice using regular microscopy. Top: representative staining images of indicated mouse testis

sections, scale bar, 20 μm; bottom, the enlargement of yellow framed regions, scale bar, 5 μm. The developmental stages of spermatocytes and spermatids were distinguished according to γH2AX (green) and DAPI (greyscale) staining. PS, pachytene spermatocytes; DS, diplotene spermatocytes; RS, round spermatids. **b** MIWI methylation and MIWI-TDRD6 interaction were not altered in *Tdrd6*$^{-/-}$ mouse. Anti-MIWI IP pellets from adult wildtype (lane 2) and *Tdrd6*$^{-/-}$ testis lysates (lane 3) were immunoblotted by the indicated antibodies, with IgG IP (lanes 1 and 3) serving as a negative control. MIWI methylation was examined by anti-SYM10 blotting. Quantification of blot intensity of indicated proteins in anti-MIWI IP pellets is shown in parentheses [the one from wildtype control mouse (lane 2) is set as 1.0 after normalization with MIWI blotting]. **c** Immunostaining of TDRD6 (red) on testis sections from adult wildtype, *Miwi*$^{YY/YY}$, *Miwi*$^{YK/YK}$ and *Miwi*$^{-/-}$ mice using regular microscopy. Top, representative staining images of indicated mouse testis sections, scale bar, 20 μm; bottom, the enlargement of yellow framed regions, scale bar, 5 μm. The developmental stages of spermatocytes and spermatids were distinguished according to γH2AX (green) and DAPI (greyscale) staining. PS, pachytene spermatocytes; DS, diplotene spermatocytes; RS, round spermatids. **d** Peanut agglutinin (PNA) staining of testis sections from wildtype, *Miwi*$^{YY/YY}$, *Miwi*$^{YK/YK}$ and *Miwi*$^{-/-}$ mice using regular microscopy, with developmental stages of the seminiferous tubules distinguished by γH2AX (green) and DAPI (grayscale) staining. Scale bar, 10 μm. Results shown are representative of three independent experiments. Quantification of western blot analysis are represented as mean ± SD. Source data are provided as a Source Data file.

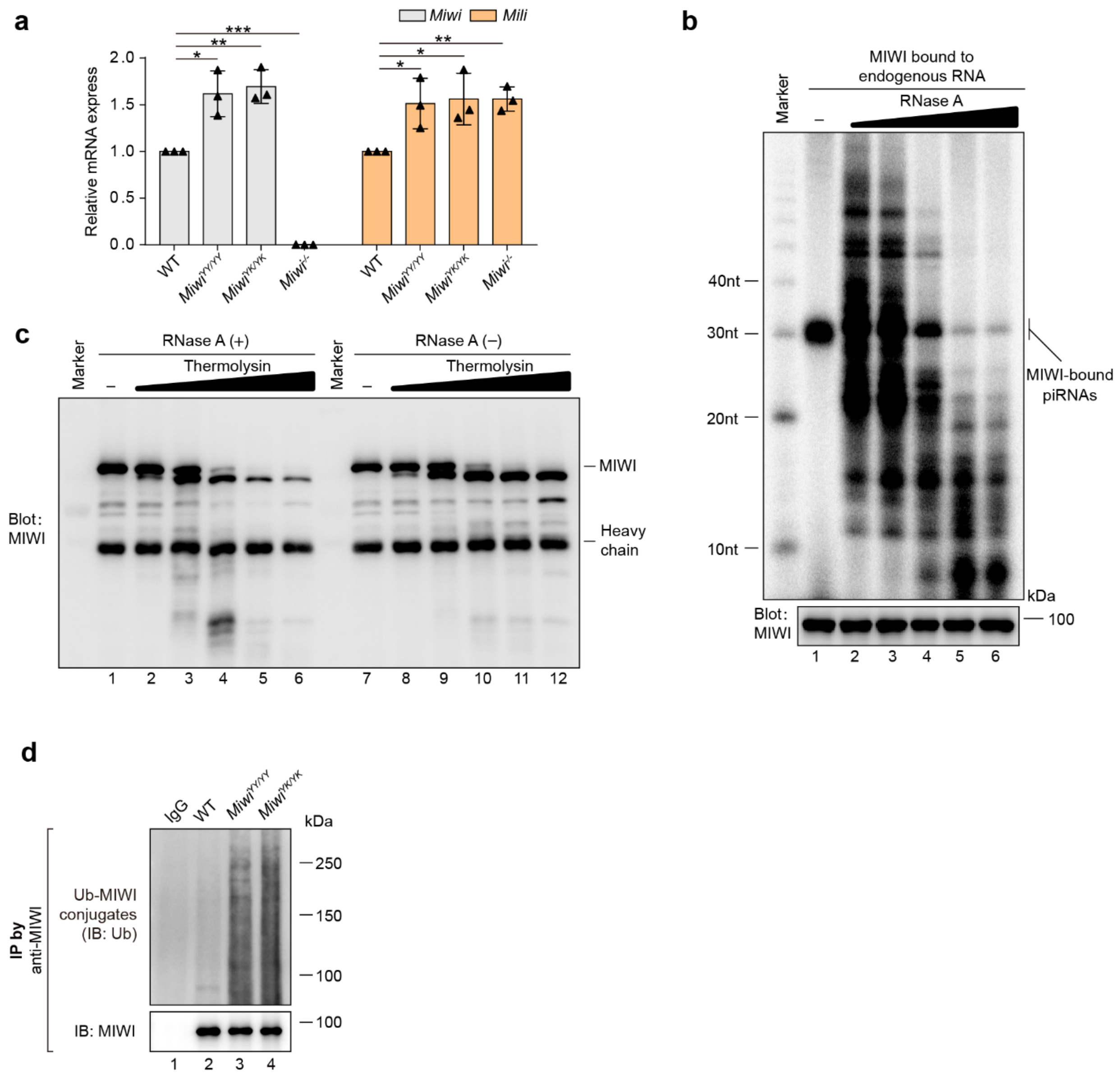

Fig. S7 | Loss of piRNA-loading ability impairs MIWI stability in mouse testes. **a** RT-qPCR analysis of *Miwi* and *Mili* mRNA expression in adult wildtype, *Miwi*$^{YY/YY}$, *Miwi*$^{YK/YK}$ and *Miwi*$^{-/-}$ testes. The one in wildtype testes is set as 1.0 after normalization with β-actin mRNA (n=3, data are represented as mean ± SD, *P* values were calculated using two-tailed Student's *t* test, *$p<0.05$, **$p<0.01$, ***$p<0.001$). **b** Generation of piRNA-unloaded MIWI protein from mouse testes. Anti-MIWI IP pellets in testicular lysates were treated with increasing amount of RNase A for generating piRNA-unloaded MIWI protein, with anti-MIWI IB serving as a loading reference (bottom). **c** Limited proteolytic digestion of RNase A-treated (lanes 1-6) or untreated-anti-MIWI pellets in testicular lysates

(lanes 7-12) with thermolysin. The reactions were carried out in the absence of thermolysin (lanes 1 and 7) or in the presence of increasing amounts of thermolysin (lanes 2-6 and 8-12) before immunoblotted using anti-MIWI antibody. **d** Ubiquitination of MIWI detected in adult wildtype, *Miwi*$^{YY/YY}$ and *Miwi*$^{YK/YK}$ mice. Top, anti-MIWI IP pellets (lanes 2-4) were immunoblotted by anti-Ub for Ub-MIWI conjugates, using IgG IP (lane 1) as a negative control. Bottom, anti-MIWI IB provided a loading reference. Results shown are representative of three independent experiments. Source data are provided as a Source Data file.

Table S1 Oligonucleotides and antibodies used in this study.

Primers used in RT qPCR		
**Target**	**Forward primer**	**Reverse primer**
Miwi	5'-CACGACGATCAGGGAGTGAC-3'	5'-GGTGGGATGTACCCAGGTTG-3'
Mili	5'-TCCAGGAGAGAGCGAGAGAG-3'	5'-CCAATTCCTTATCTTTCCACCACG-3'
β-actin	5'-TTACCAACTGGGACGACATG-3'	5'-AGGGACAGCACAGCCTGGAT-3'

piRNA used in *in vitro* loading assay	
Cy5-piR-1	5′-pUGACAUGAAC$_{cy5}$ACAGGUGCUCAGAUAGCUUUm-3′

Antibodies		
**Antibodies**	**Source**	**Identifier**
Rabbit polyclonal anti-MIWI	Customized in ABclonal Technology, against 250-550 aa (used for WB)	N/A, WB: 1: 1000
Rabbit polyclonal anti-MIWI	Customized in ABclonal Technology, against 1-160 aa (used for IP)	N/A, IP: 1:50
Rabbit monoclonal anti-MIWI	ABclonal	Cat# A3490; RRID: AB_2863072, IP: 1: 50
Rabbit polyclonal anti-MIWI	Cell Signaling Technology (used for IF)	Cat# 2079; RRID: AB_2165432, IF: 1: 100
Rabbit monoclonal anti-HA	Cell Signaling Technology	Cat# C29F4; RRID: AB_1549585, WB: 1:1000
Rabbit polyclonal anti-MILI	MBL	Cat# PM044; RRID: AB_1279201, IF: 1:100
Rabbit polyclonal anti-TDRKH	Proteintech	Cat# 13528-1-AP; RRID: AB_2303299, IF: 1:200, WB: 1:1000
Mouse FITC-conjugated anti-γH2AX	Millipore	Cat# 16-202A; RRID: AB_568825, IF: 1: 500
Rabbit polyclonal anti-IgG	Millipore	Cat# 12-370; RRID: AB_145841, IP: 1:50
Rabbit polyclonal anti-SYM10	Millipore	Cat# 07-412; RRID: AB_310594, WB: 1: 1000
Rabbit monoclonal anti-MVH	Abcam	Cat# ab13840; RRID: AB_443012, IF: 1: 200
Mouse monoclonal anti-β-actin	Sigma	Cat# A3854; RRID: AB_262011, WB: 1: 10000
Mouse monoclonal anti-Flag	Sigma	Cat# F3165; RRID: AB_259529, WB: 1: 1000
Rabbit polyclonal anti-TDRD6	Homemade	N/A, IF: 1: 200
Rabbit monoclonal anti-GFP	Beyotime	Cat# AF1483; Ref: PMID30241539, WB: 1: 1000
Donkey anti-Rabbit IgG (Alexa Fluor 555)	ThermoFisher	Cat# A31572; RRID: AB_162543, IF: 1: 500
Goat anti-Rabbit IgG (HRP conjugate)	Sigma	Cat# A9169; RRID: AB_258434, WB: 1: 10000
Goat anti-Mouse IgG (HRP conjugate)	Sigma	Cat# A0168; RRID: AB_257867, WB: 1: 10000
Rabbit monoclonal anti-Ubiquitin	Huabio	Cat# ET1609-21; RRID: AB_3069833, WB: 1: 1000
Mouse monoclonal anti-Cytochrome c	Proteintech	Cat# 66264-1-Ig; RRID: AB_2716798, IF: 1: 100
Rabbit polyclonal anti-ACRV1	Proteintech	Cat# 14040-1-AP; RRID: AB_10640426, IF: 1: 50
