# Supplementary material for: piRNA loading triggers MIWI translocation from the intermitochondrial cement to chromatoid body during mouse spermatogenesis
Source: Nat Commun. 2024 Mar 15;15:2343. doi: 10.1038/s41467-024-46664-3 (PMC10943014; doi:10.1038/s41467-024-46664-3)
Supplement: Supplementary file 3 — Reporting Summary [file 41467_2024_46664_MOESM3_ESM.pdf]

nature portfolio

Corresponding author(s): Mo-Fang Liu; Deqiang Ding; Xin Wang

Last updated by author(s): Feb 7, 2024

Reporting Summary

Nature Portfolio wishes to improve the reproducibility of the work that we publish. This form provides structure for consistency and transparency in reporting. For further information on Nature Portfolio policies, see our Editorial Policies and the Editorial Policy Checklist.

Statistics

For all statistical analyses, confirm that the following items are present in the figure legend, table legend, main text, or Methods section.

n/a	Confirmed	
☐	☒	The exact sample size (*n*) for each experimental group/condition, given as a discrete number and unit of measurement
☐	☒	A statement on whether measurements were taken from distinct samples or whether the same sample was measured repeatedly
☐	☒	The statistical test(s) used AND whether they are one- or two-sided<br>*Only common tests should be described solely by name; describe more complex techniques in the Methods section.*
☒	☐	A description of all covariates tested
☐	☒	A description of any assumptions or corrections, such as tests of normality and adjustment for multiple comparisons
☐	☒	A full description of the statistical parameters including central tendency (e.g. means) or other basic estimates (e.g. regression coefficient) AND variation (e.g. standard deviation) or associated estimates of uncertainty (e.g. confidence intervals)
☐	☒	For null hypothesis testing, the test statistic (e.g. *F*, *t*, *r*) with confidence intervals, effect sizes, degrees of freedom and *P* value noted<br>*Give P values as exact values whenever suitable.*
☒	☐	For Bayesian analysis, information on the choice of priors and Markov chain Monte Carlo settings
☒	☐	For hierarchical and complex designs, identification of the appropriate level for tests and full reporting of outcomes
☒	☐	Estimates of effect sizes (e.g. Cohen's *d*, Pearson's *r*), indicating how they were calculated

Our web collection on statistics for biologists contains articles on many of the points above.

Software and code

Policy information about availability of computer code

Data collection	Tanon FLICapture (5200 chemiluminescent imaging system) was used to collect western blot image data and the average values of band intensity were calculated using Image-Pro Plus 6.0 software. CKX53 fluorescence 414 microscope (Olympus, Japan) or Ti2-E confocal microscope (Nikon, Japan) was used to collect immunohistochemical image data. Thermofisher QuantStudio 3 Real-Time PCR System was used to collect qPCR data. FLA-9000 image analyzer (Fujifilm) was used to collect autoradiography image data.
Data analysis	Graphpad_Prism7, Photoshop CS3, Adobe Illustrator CC 2017, Image-J 1.47v, R v3.6.3, Python packages MDTraj 1.9.4.

For manuscripts utilizing custom algorithms or software that are central to the research but not yet described in published literature, software must be made available to editors and reviewers. We strongly encourage code deposition in a community repository (e.g. GitHub). See the Nature Portfolio guidelines for submitting code & software for further information.

Data

Policy information about availability of data

All manuscripts must include a data availability statement. This statement should provide the following information, where applicable:

- Accession codes, unique identifiers, or web links for publicly available datasets
- A description of any restrictions on data availability
- For clinical datasets or third party data, please ensure that the statement adheres to our policy

All data presented are available in the main text and supplementary materials. The small RNA-seq data used in this study are available in the BioProject field of

National Center for Biotechnology Information (NCBI) under accession code PRJNA979781 (https://www.ncbi.nlm.nih.gov/bioproject/?term=PRJNA979781). The RepeatMasker (ftp://hgdownload.cse.ucsc.edu/goldenPath/mm10/database/rmsk.txt.gz) was used to define the category "repeats". Source data are provided with this paper.

Research involving human participants, their data, or biological material

Policy information about studies with human participants or human data. See also policy information about sex, gender (identity/presentation), and sexual orientation and race, ethnicity and racism.

Reporting on sex and gender	N/A
Reporting on race, ethnicity, or other socially relevant groupings	N/A
Population characteristics	N/A
Recruitment	N/A
Ethics oversight	N/A

Note that full information on the approval of the study protocol must also be provided in the manuscript.

Field-specific reporting

Please select the one below that is the best fit for your research. If you are not sure, read the appropriate sections before making your selection.

☒ Life sciences ☐ Behavioural & social sciences ☐ Ecological, evolutionary & environmental sciences

For a reference copy of the document with all sections, see nature.com/documents/nr-reporting-summary-flat.pdf

Life sciences study design

All studies must disclose on these points even when the disclosure is negative.

Sample size	Statistical methods were not employed for sample size prediction; instead, we used a generally accepted sample size based on our laboratory's prior experience (PMID: 32017896, 31835033). The reproducibility differences observed among samples due to mutations suggest that our chosen sample size was adequate. Our sample size is consistent with those commonly used in the life sciences. All experiments were conducted with at least three independent biological replicates under each condition.
Data exclusions	No data were excluded from analyses.
Replication	Unless explicitly stated, all data shown were obtained from 3 or more biological independent experiments with technical replicates. All replications were successful, and the detailed information was provided in corresponding figure legends.
Randomization	All C57BL/6J mice were randomly divided into the control and experimental groups. The cells were randomized into different groups prior to treatment with MTA or RNase A. The transgenic mice (WT, MiwiYY/YY, MiwiYK/YK, Miwi-/-, and Tdrd6-/-) were allocated based on the results of PCR genotyping performed two weeks after birth.
Blinding	No blinding was performed since there was no specific grouping.

Reporting for specific materials, systems and methods

We require information from authors about some types of materials, experimental systems and methods used in many studies. Here, indicate whether each material, system or method listed is relevant to your study. If you are not sure if a list item applies to your research, read the appropriate section before selecting a response.

Materials & experimental systems

n/a	Involved in the study
☐	☒ Antibodies
☐	☒ Eukaryotic cell lines
☒	☐ Palaeontology and archaeology
☐	☒ Animals and other organisms
☒	☐ Clinical data
☒	☐ Dual use research of concern
☒	☐ Plants

Methods

n/a	Involved in the study
☒	☐ ChIP-seq
☒	☐ Flow cytometry
☒	☐ MRI-based neuroimaging

Antibodies

Antibodies used

Antibodies used in western blotting:
Rabbit polyclonal anti-MIWI, Customized in ABclonal Technology, against 250-550 aa (used for WB), N/A, 1: 1000
Rabbit polyclonal anti-TDRKH, Proteintech, Cat# 13528-1-AP; RRID: AB_2303299, 1: 1000
Rabbit monoclonal anti-HA, Cell Signaling Technology, Cat# C29F4; RRID: AB_1549585, 1: 1000
Rabbit polyclonal anti-SYM10, Millipore, Cat# 07-412; RRID: AB_310594, 1: 1000
Mouse monoclonal anti-β-actin, Sigma, Cat# A3854; RRID: AB_262011, 1: 10000
Mouse monoclonal anti-Flag, Sigma, Cat# F3165; RRID: AB_259529, 1: 1000
Rabbit monoclonal anti-GFP, Beyotime, Cat# AF1483; Ref: PMID30241539, 1: 1000
Goat anti-Rabbit IgG (HRP conjugate), Sigma, Cat# A9169; RRID: AB_258434, 1: 10000
Goat anti-Mouse IgG (HRP conjugate), Sigma, Cat# A0168; RRID: AB_257867, 1: 10000
Rabbit monoclonal anti- Ubiquitin, Huabio, Cat# ET1609-21; RRID: AB_3069833, 1: 1000

Antibodies used in immunofluorescent staining:
Rabbit polyclonal anti-ACRV1, Proteintech, Cat# 14040-1-AP; RRID: AB_10640426, 1: 50
Rabbit polyclonal anti-TDRKH, Proteintech, Cat# 13528-1-AP; RRID: AB_2303299, 1: 200
Rabbit polyclonal anti-MIWI, Cell Signaling Technology (used for IF), Cat# 2079; RRID: AB_2165432, 1: 100
Rabbit polyclonal anti-MILI, MBL, Cat# PM044; RRID: AB_1279201, 1: 100
Rabbit monoclonal anti-MVH, Abcam, Cat# ab13840; RRID: AB_443012, 1:200
Rabbit polyclonal anti-TDRD6, Homemade, N/A, 1:200
Mouse FITC-conjugated anti-γH2AX, Millipore, Cat# 16-202A; RRID: AB_568825, 1: 500
Donkey anti-Rabbit IgG (Alexa Fluor 555), ThermoFisher, Cat# A31572; RRID: AB_162543, 1: 500
Mouse monoclonal anti-Cytochrome c, Proteintech, Cat# 66264-1-Ig; RRID: AB_2716798, 1: 100

Antibodies used in co-immunoprecipitation:
Rabbit polyclonal anti-MIWI, Customized in ABclonal Technology, against 1-160 aa (used for IP), N/A, 1:50
Rabbit monoclonal anti-MIWI, ABclonal, Cat# A3490; RRID: AB_2863072, 1: 50
Rabbit polyclonal anti-IgG, Millipore, Cat# 12-370; RRID: AB_145841, 1: 50

Validation

Apart from a few homemade antibodies, all other antibodies used in this study were commercially available and were validated by the manufactures, validation statements are available on the manufacturer's website.

Rabbit monoclonal anti-GFP: tested applications suitable for: WB 1:1000-1:5000, IP 1:10-1:50, IF/ ICC 1:50-1:200. (https://www.beyotime.com/product/AF1483.htm, Ref: PMID: 30241539)

Eukaryotic cell lines

Policy information about cell lines and Sex and Gender in Research

Cell line source(s)

Female Human Embryonic Kidney 293T (HEK293T) cells (ATCC, CRL-3216) and male mouse spermatocyte-derived GC-2spd (ts) cells (ATCC, CRL-2196).

Authentication

Authentication was not performed as none of the cells we used have been listed in the commonly misidentified lines.

Mycoplasma contamination

HEK293T and GC-2spd(ts) were tested negative for mycoplasma contamination.

Commonly misidentified lines
(See ICLAC register)

No commonly misidentified cell line was used in this study.

Animals and other research organisms

Policy information about studies involving animals; ARRIVE guidelines recommended for reporting animal research, and Sex and Gender in Research

Laboratory animals

Male C57BL/6J mice (WT, MiwiYY/YY, MiwiYK/YK, Miwi-/-, and Tdrd6-/-) at 18-dpp, 20-dpp, 24-dpp or 6-8 weeks (adult) of age were

Laboratory animals	used in this study. All mice were on the C57BL/6J genetic background. Mice were housed under 12-hour light/dark cycles in a pathogen-free room with clean bedding and free access to food and water, and temperature and humidity were kept at 23 ± 1°C, 55 ± 5%. Cage and bedding changes were performed each week.
Wild animals	No wild animal was used.
Reporting on sex	This study focused on spermatogenesis, and the findings applied to only male mice.
Field-collected samples	No field-collected sample was used.
Ethics oversight	All mice were housed in the SIBCB animal facility under SPF conditions in accordance with institutional guidelines and ethical regulations, and fed with regular chow and water by the facility staff. All experimental animal procedures were approved by the Institutional Animal Care and Research Advisory Committee at SIBCB, CAS (2022-046).

Note that full information on the approval of the study protocol must also be provided in the manuscript.
