# Supplementary material for: piRNA loading triggers MIWI translocation from the intermitochondrial cement to chromatoid body during mouse spermatogenesis
Source: Nat Commun. 2024 Mar 15;15:2343. doi: 10.1038/s41467-024-46664-3 (PMC10943014; doi:10.1038/s41467-024-46664-3)

Figure S2a

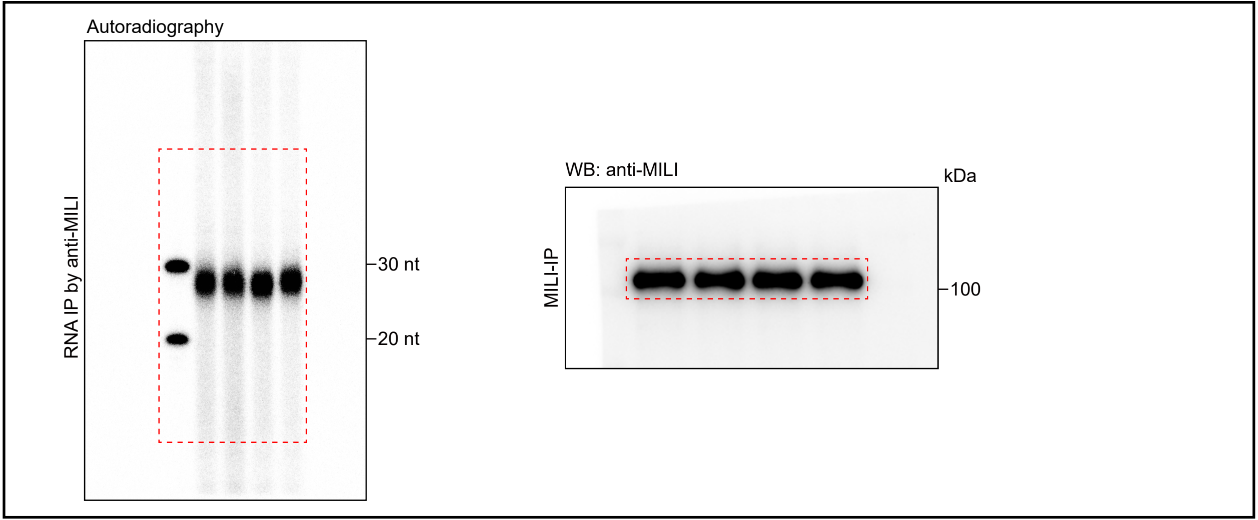
Autoradiography
RNA IP by anti-MILI
30 nt
20 nt
WB: anti-MILI
kDa
MILI-IP
100

Figure S4a

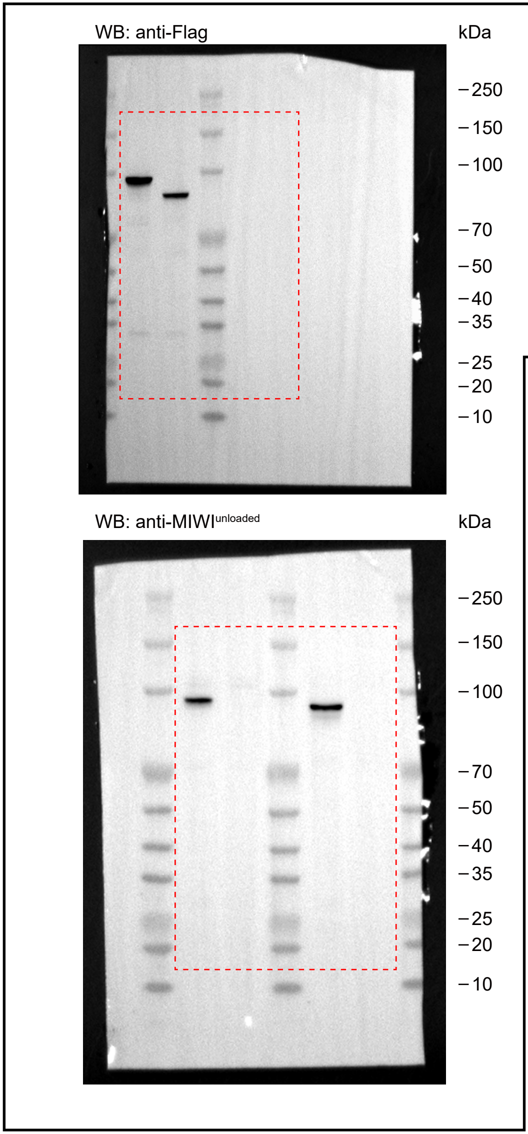

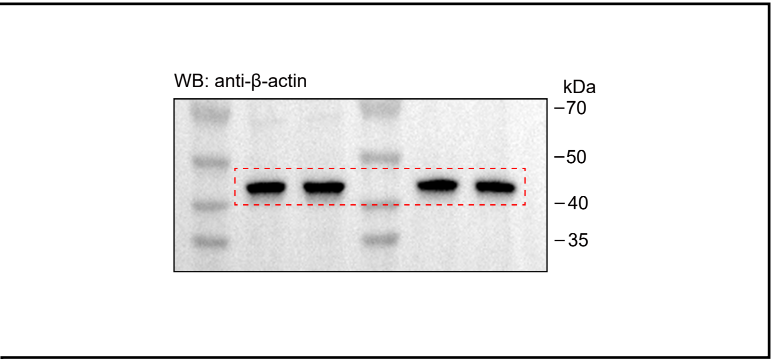

Figure S4b

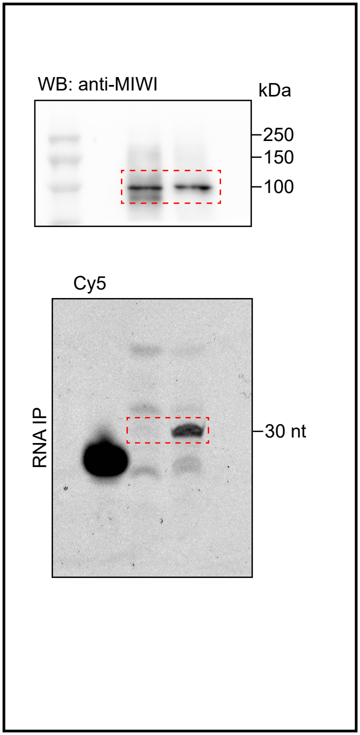

Figure S4c

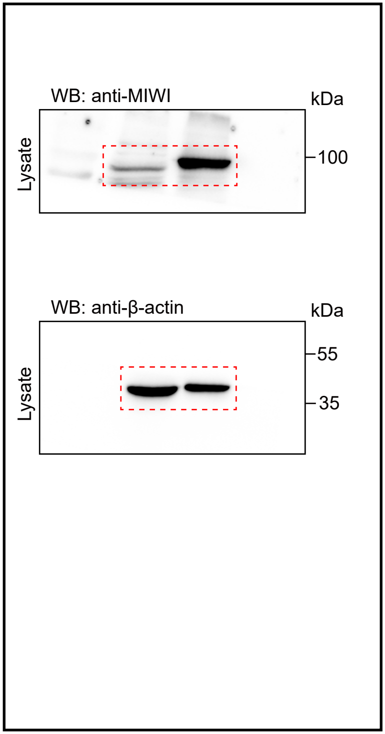

Figure S5d

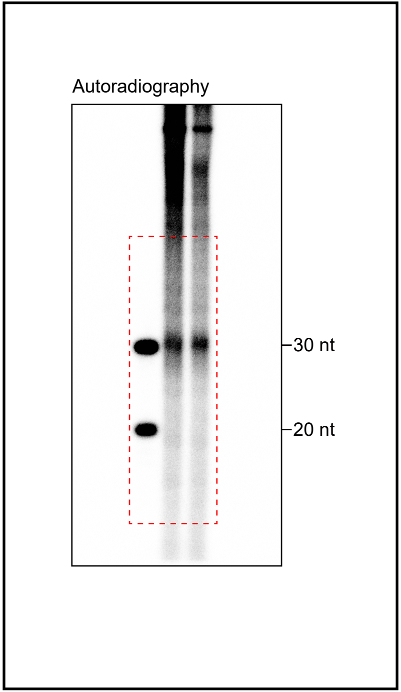

Figure S5i

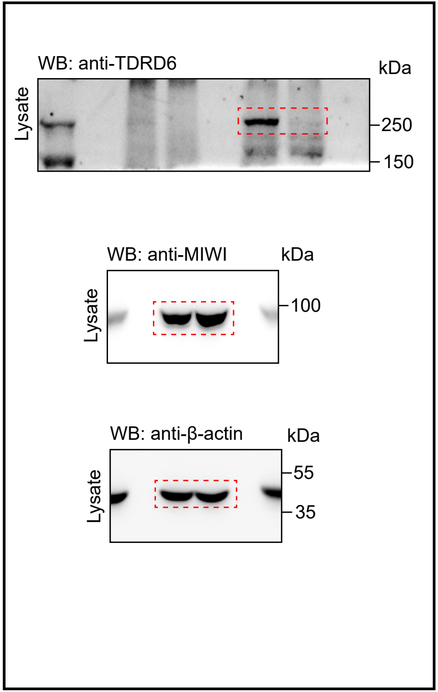

Figure S5j

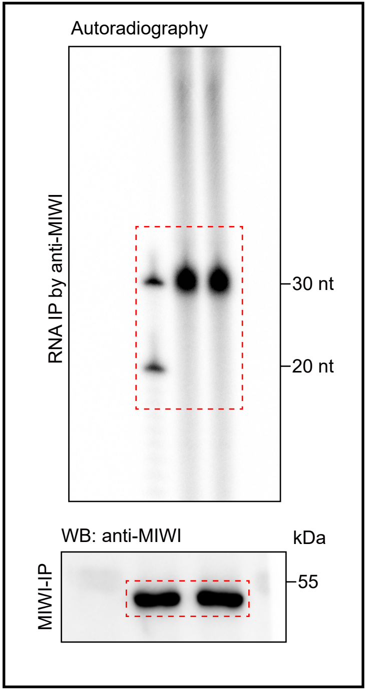

Figure S6b

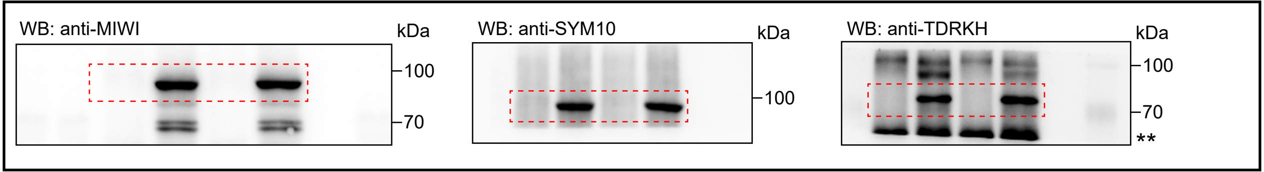
WB: anti-MIWI
kDa
100
70
WB: anti-SYM10
kDa
100
WB: anti-TDRKH
kDa
100
70
**

Figure S7b

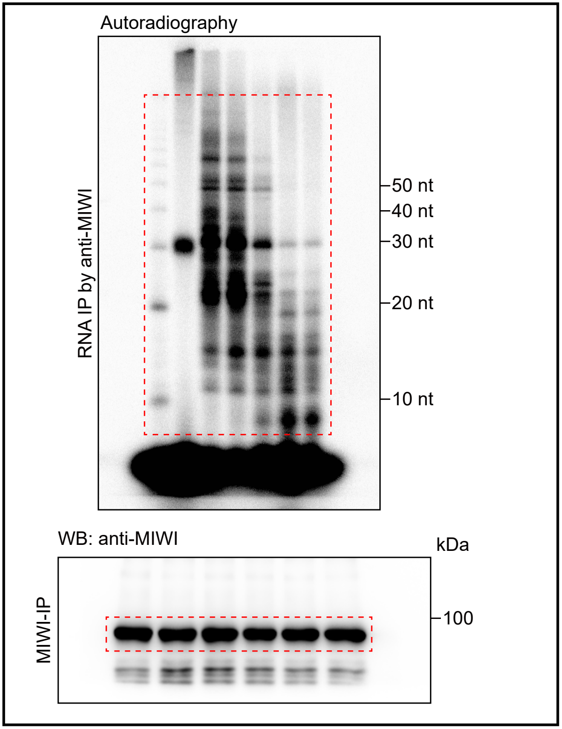

Figure S7c

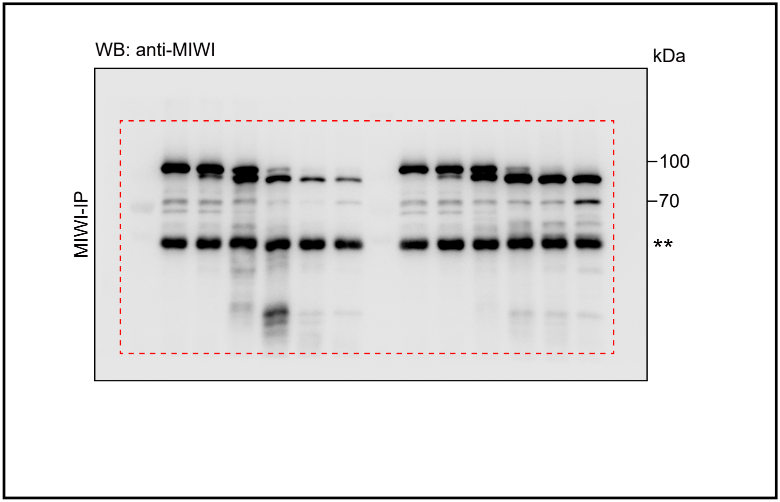

Figure S7d

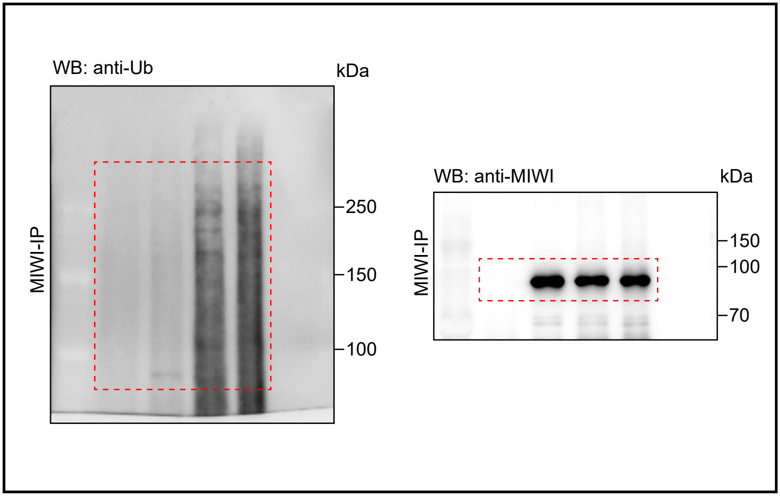

Figure S7e

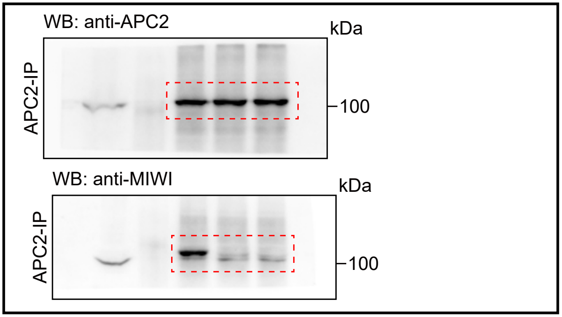

Supplement: Supplementary file 4 — Source Data [file 41467_2024_46664_MOESM4_ESM.zip › Source data_Supplemental Figures 1-7 blots and gels.pdf]
