# Supplementary material for: piRNA loading triggers MIWI translocation from the intermitochondrial cement to chromatoid body during mouse spermatogenesis
Source: Nat Commun. 2024 Mar 15;15:2343. doi: 10.1038/s41467-024-46664-3 (PMC10943014; doi:10.1038/s41467-024-46664-3)

Figure 1b

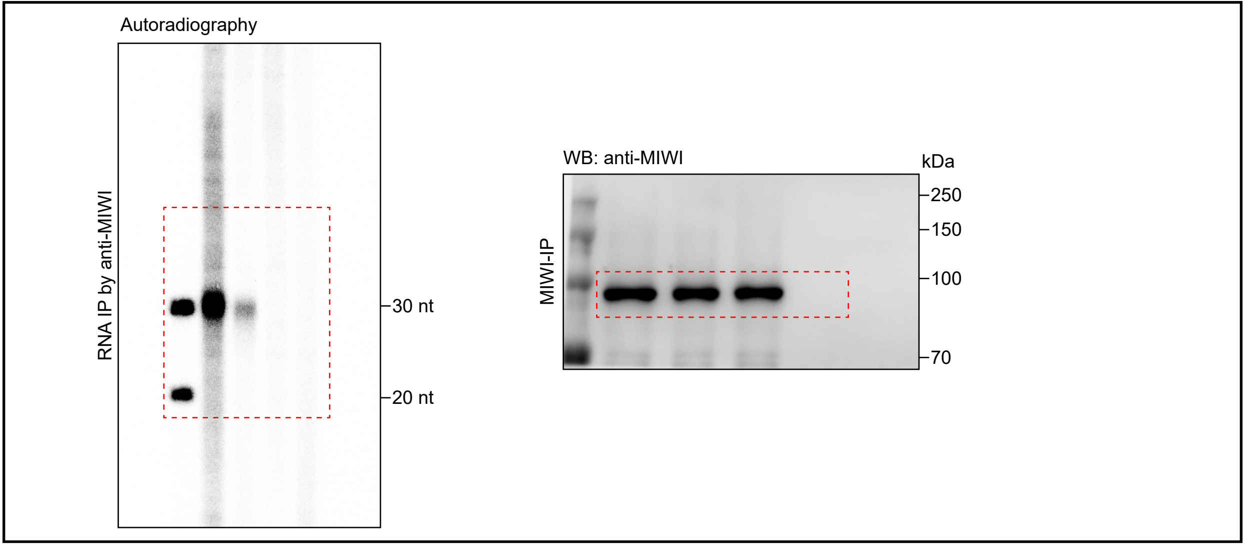

Autoradiography
RNA IP by anti-MIWI
30 nt
20 nt
WB: anti-MIWI
kDa
MIWI-IP
250
150
100
70

Figure 2a

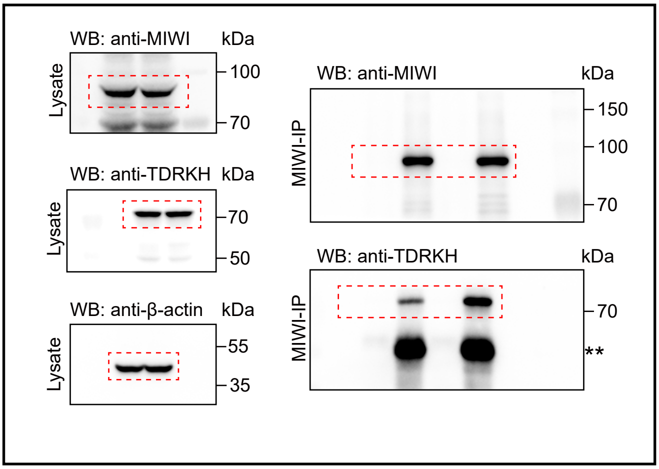

Figure 2b

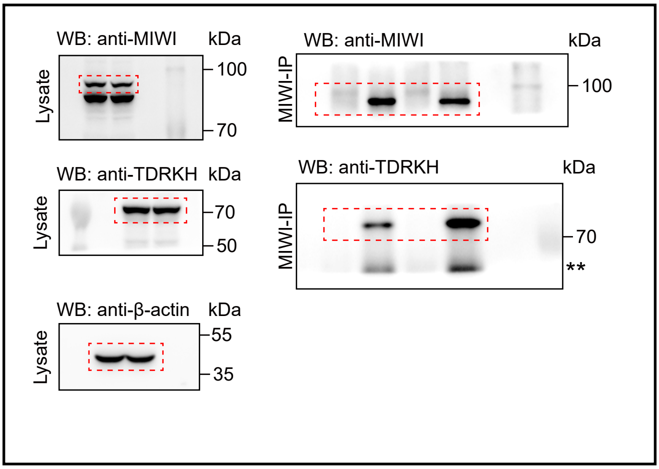

Figure 2c

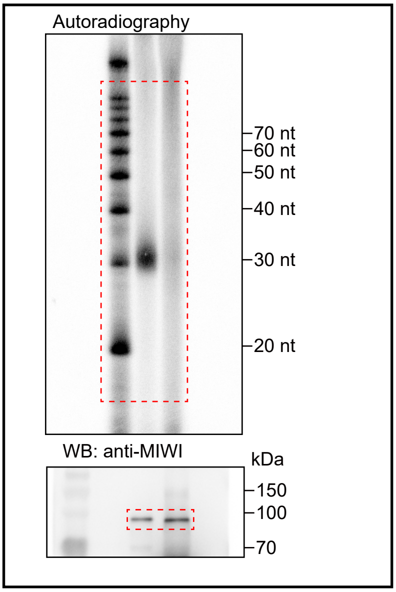

Figure 2d

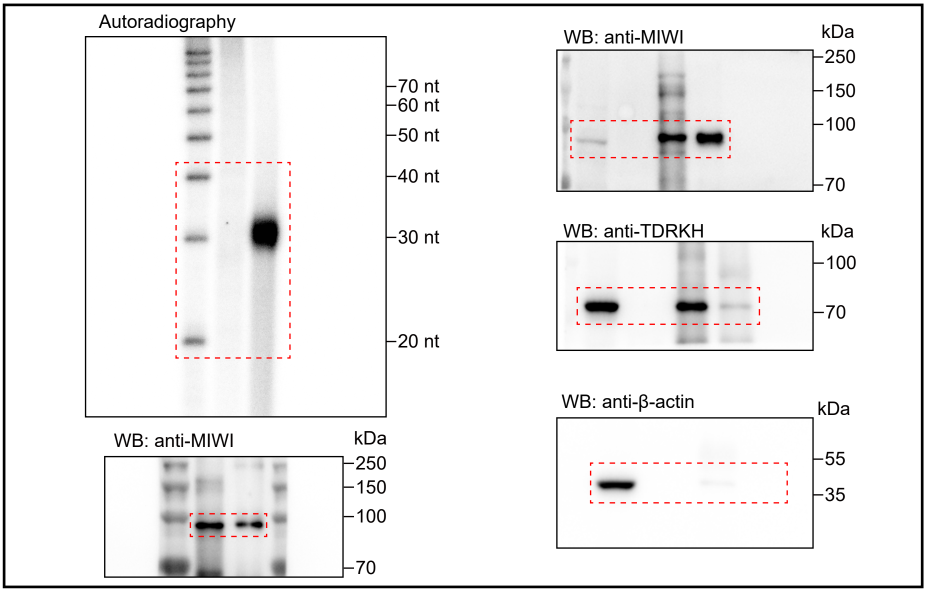

Figure 2e

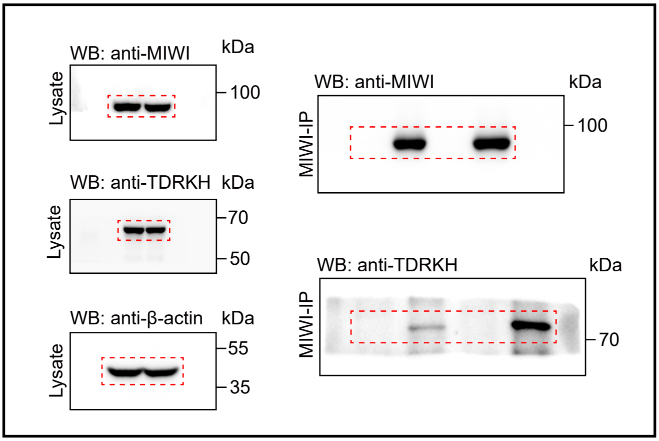

Figure 2f

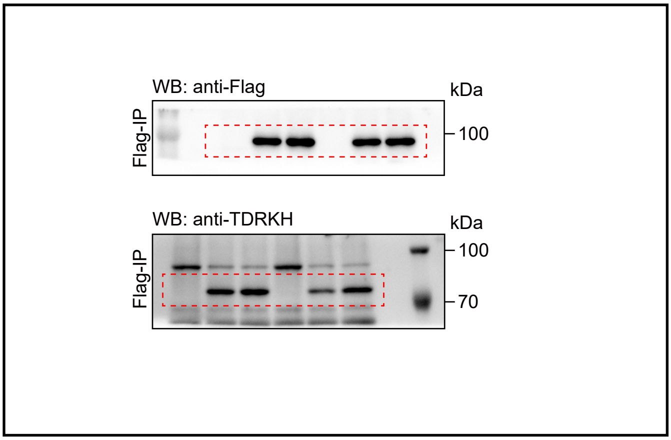

Figure 3c

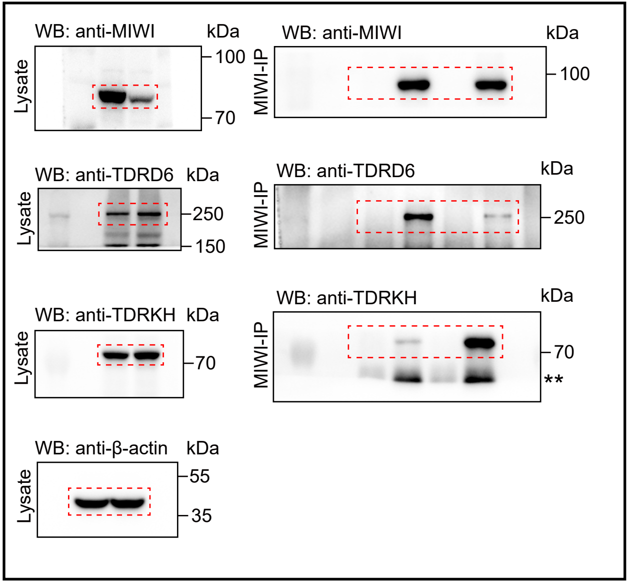

Figure 3d

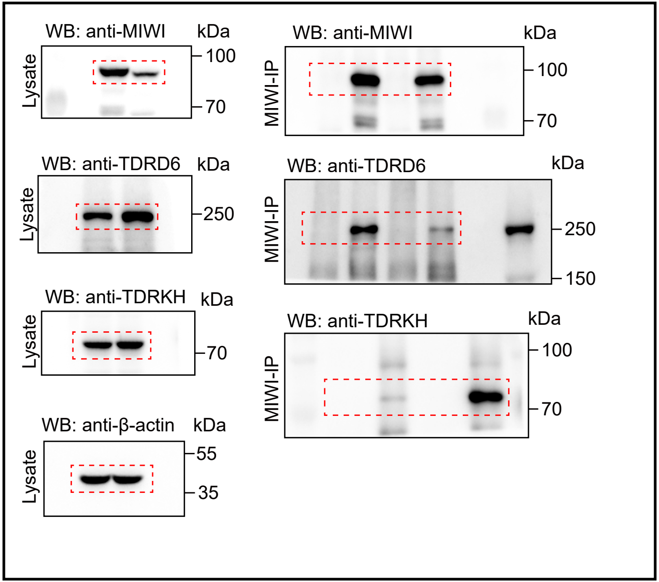

Figure 4a

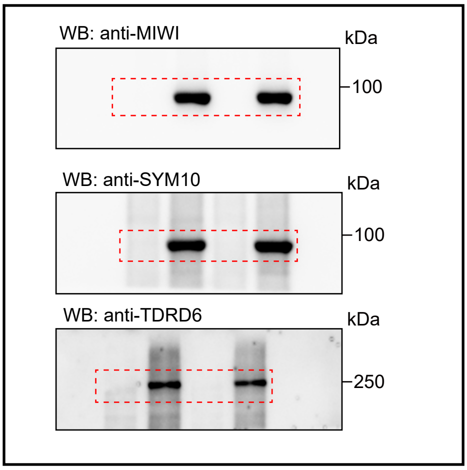

Figure 4b

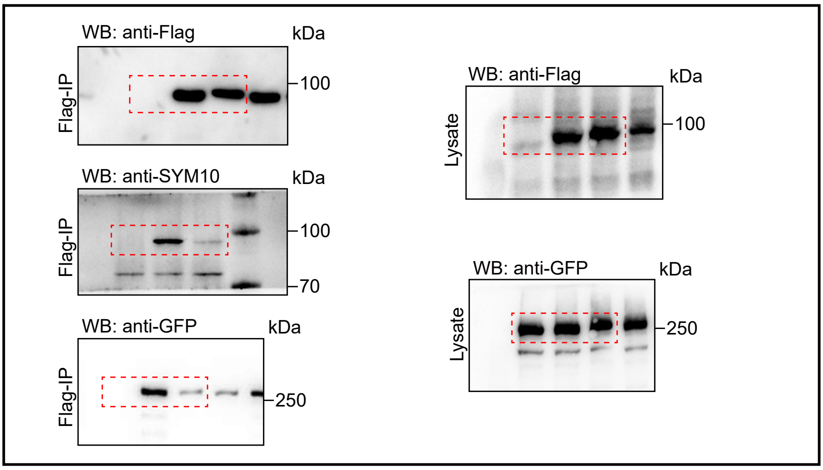

Figure 4c

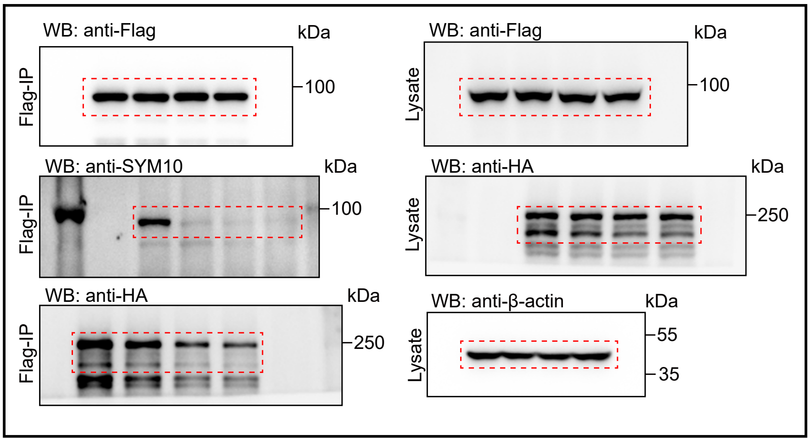

Figure 4d

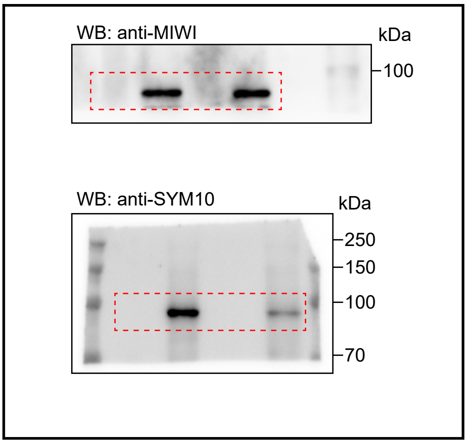

Figure 4e

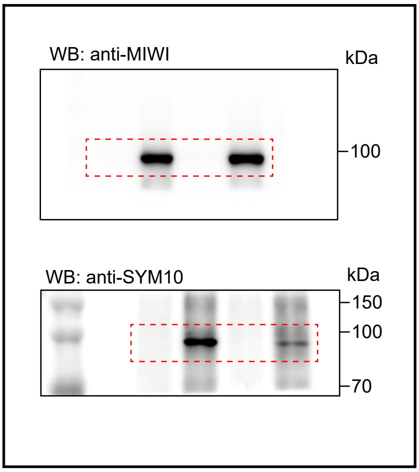

Figure 4f

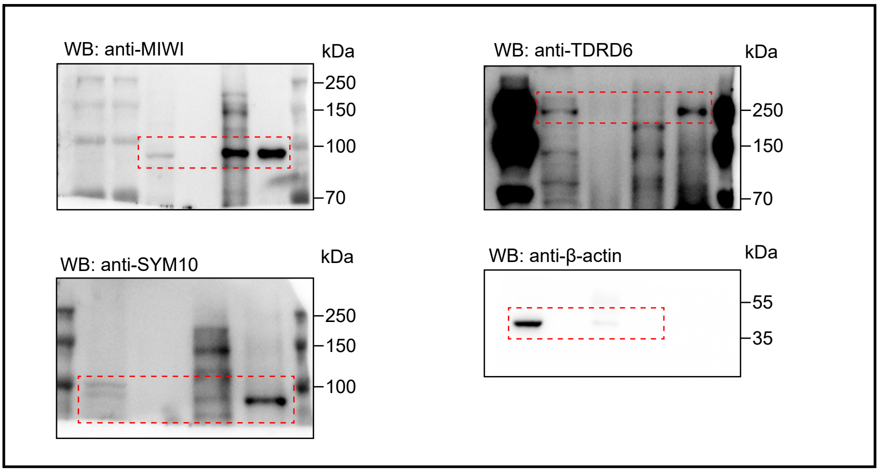

Figure 4g

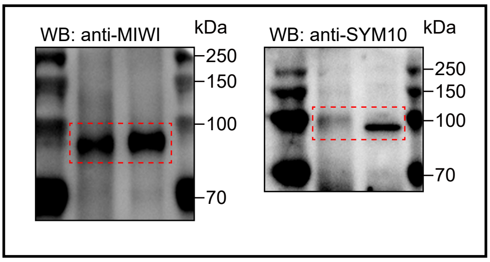

Figure 4h

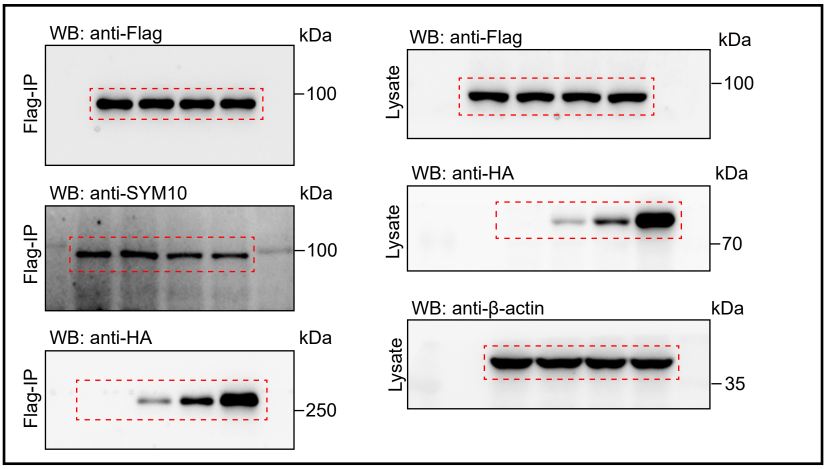

Figure 6a

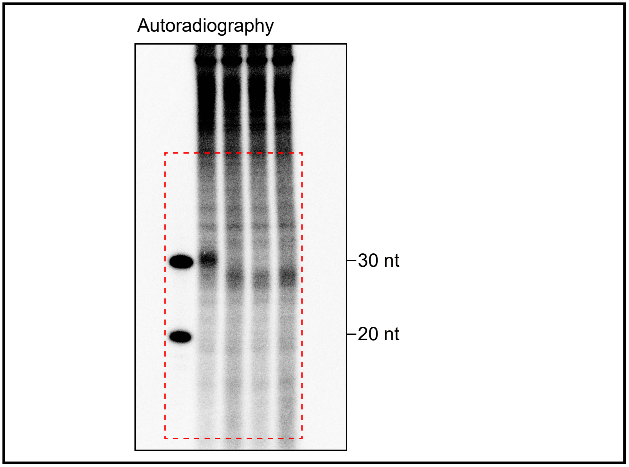

Figure 6f

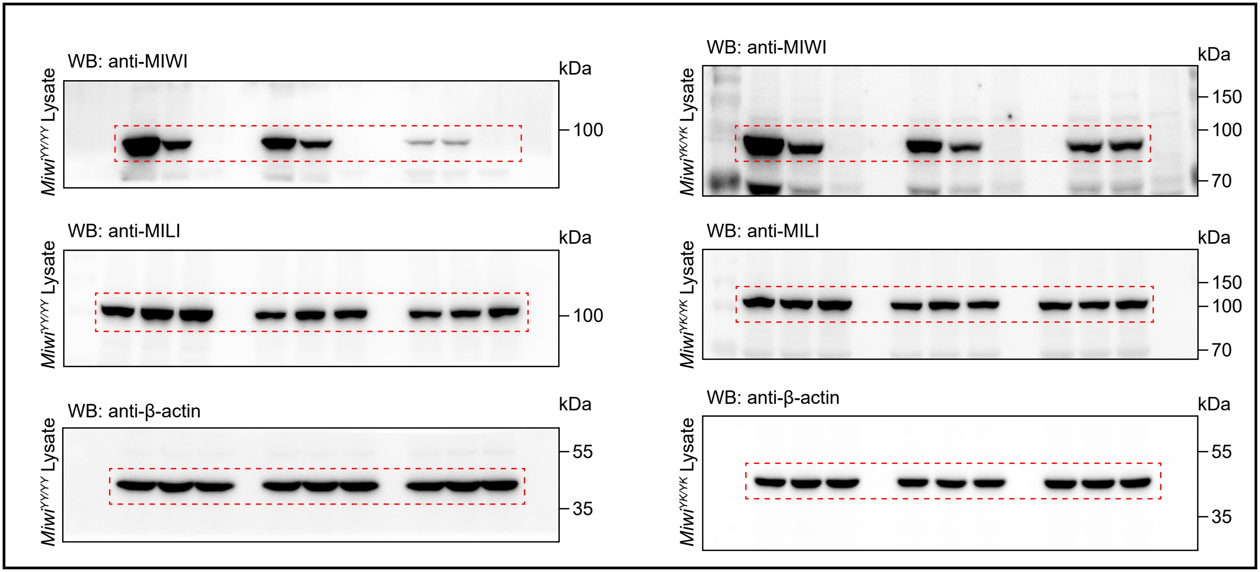

Supplement: Supplementary file 4 — Source Data [file 41467_2024_46664_MOESM4_ESM.zip › Source data_Figures 1-7 blots and gels.pdf]
